# Social-pair judgment bias testing in slow-growing broiler chickens raised in low- or high-complexity environments

**DOI:** 10.1038/s41598-023-36275-1

**Published:** 2023-06-09

**Authors:** M. I. Lourenço-Silva, A. Ulans, A. M. Campbell, I. C. L. Almeida Paz, L. Jacobs

**Affiliations:** 1 Department of Animal Production and Preventive Veterinary Medicine, School of Veterinary Medicine and Animal Sciences (FMVZ), São Paulo State University “Júlio de Mesquita Filho” (UNESP), Botucatu, São Paulo Brazil; 2 School of Animal Sciences, Virginia Tech, Blacksburg, VA 24061 USA

**Keywords:** Animal behaviour, Zoology

## Abstract

Impacts of environmental complexity on affective states in slow-growing broiler chickens (*Gallus gallus domesticus*) are unknown. Chickens’ performance in judgment bias tests (JBT) can be limited as they are tested individually, causing fear and anxiety. The objectives were to apply a social-pair JBT to assess the effect of environmental complexity on slow-growing broiler chickens` affective states, and assess the impact of fearfulness, anxiety, and chronic stress on JBT performance. Six-hundred Hubbard Redbro broilers were housed in six low-complexity (similar to commercial) or six high-complexity (permanent and temporary enrichments) pens. Twelve chicken pairs were trained (1 pair/pen, n = 24 chickens) using a multimodal approach (visual and spatial cues), with reward and neutral cues of opposing color and location. Three ambiguous cues were tested: near-positive, middle, and near-neutral cues. Approach and pecking behavior were recorded. Eighty-three percent of chickens (20/24) were successfully trained in 13 days. Fearfulness, anxiety, and chronic stress did not impact chickens’ performance. Chickens successfully discriminated between cues. Low-complexity chickens approached the middle cue faster than high-complexity chickens, indicating that they were in a more positive affective state. The environmental complexity provided in this study did not improve affective states in slow-growing broiler chickens compared to a control. A social-pair JBT resulted in excellent learning and testing outcomes in slow-growing broilers.

## Introduction

Positive affective states in chickens can been assessed through the use of cognitive bias tests^1,2^. This sort of assessment can provide insights in affect-induced judgment, attention, or memory bias^3^. Judgment bias testing (JBT) is used to determine levels of optimism and pessimism of individuals based on their responses to ambiguous cues during testing^1^. Generally, this test shows good validity as determined through corroborating methods of affective state assessment. However, JBT findings can be counter-intuitive and exhibit high levels of individual variability^2^.

The JBT has been applied to study chickens’ affective states using different testing designs and conditions, such as Go/Go and Go/No-Go tasks to assess the effects of environmental conditions^4–7^, feather pecking genetic selection^8^, impact of corticosterone injections^9^, pharmacological reversal and an anxiety-depression model^10,11^, temperature manipulation^12^, and acute stress^13^. Chickens showed sensibility to distinct conditions and presented more pessimistic judgments when raised in a non-stimulating environment^4,5^, when injected with high corticosterone levels^9^, or when pharmaceutically induced to be anxious and depressed^10,11,13^. Unexpectedly, high feather pecking lines and acutely stressed hens approached ambiguous cues faster, suggesting optimism associated with these negative states^8,13^. In other studies, a complex environment did not induce a more positive affective state^4,7^. These unexpected outcomes could be associated with testing methodology, as chickens, a social species, were trained and tested individually^4–9,12,13^.

The gregarious nature of chickens could potentially be utilized when training and performing the JBT. Social interaction and environment can shape cognition and vice versa^14^. Chickens are influenced by social factors that mediate learning and memory processes^15^. For instance, imprinting behavior and social facilitation stimulate learning^15–18^. Similarly, social isolation during early development provoked distress and hampered spatial learning in adult chickens^19^, and social isolation for five or sixty minutes resulted in pessimistic judgments in a JBT^10,11^. In addition, chronic distress can negatively impact cognitive ability associated with learning by shaping neuronal dendritic morphology and decreasing dendritic complexity within the hippocampus^20^. For instance, chronically distressed rodents showed behaviors associated with anhedonia and decreased motivation, impairing their spatial acquisition in appetitively motivated tasks^21–24^ similar to a JBT. Thus, a social training and testing approach could potentially improve learning and performance directly through social facilitation, and indirectly through reduced distress.

Individual preferences, fearfulness, and anxiety can impact chickens’ learning processes and outcomes in a JBT^25,26^. Prior studies trained and tested birds individually, and training success varied^5–7,9,12,13^. A social approach, where learning is stimulated, could improve training and testing outcomes. Laying hens with a reactive behavioral response learned JBT tasks quicker during training, while fearful and stress-sensitive hens developed side biases and did not meet the JBT training criteria^26^. This suggests that these behavioral traits should be accounted for when performing a JBT study. Social experiences and intrinsic state, i.e. food competition, hierarchy, environmental conditions, individual levels of fat reserves, metabolic rate, and hormone levels shape behavioral responses and individual preferences^27^. This further suggests that individual characteristics (fearfulness, anxiety, and social experiences) should be considered when performing cognitive bias tests.

A monotonous environment common in conventional fast-growing broiler chicken houses does not favor the expression of natural behaviors, i.e. foraging, dustbathing, and perching, which can improve animal welfare and cognition^5,28–30^. Providing enrichments increases the environmental complexity and the expression of these natural behaviors in fast-growing broiler chickens^31–33^, and improves aspects of affective state^5^. A similar response may be observed in slower growing broiler chicken strains.

The effects of environmental enrichment on the affective states of slow-growing broiler chickens, and the potential benefit of a social JBT approach have not been examined. Therefore, this study aimed to assess the affective states of slow-growing broiler chickens raised in high- or low-complexity environments, using a novel social-pair JBT approach. We also aimed to evaluate the effects of fearfulness, anxiety, and chronic stress (feather corticosterone) on training and testing performance. We hypothesized that chickens from high-complexity environments would make more optimistic choices than chickens from low-complexity environments. Additionally, we hypothesized that a social-pair approach would attenuate fearfulness, anxiety, and chronic stress effects, resulting in improved learning and thus judgment bias training performance. Improved learning would be reflected in fewer training rounds needed to meet the training criteria.

## Methods

### Ethics declarations

The trial was carried out at Virginia Tech’s Turkey Research Center from March through May 2022. Virginia Tech’s Institutional Animal Care and Use Committee approved the experimental protocol as part of a larger experiment (protocol number 21-221). The experiment was performed following relevant guidelines and regulations. This study is reported in accordance with ARRIVE guidelines^34^.

### Animals and housing

Six hundred day-old male Hubbard Redbro (slow-growing strain) broiler chicks from a commercial hatchery where they were vaccinated for Marek’s disease, followed by 6-h transportation to the research facility. The trial was carried out in a fully automated climate-controlled poultry house with negative pressure ventilation. Chicks were randomly allocated to 12 pens of 8.75 m^2^ each, with 50 chicks per pen. Calculated stocking densities at 22, 44, and 53 days of age were 3.88 ± 0.06, 12.14 ± 0.28, and 16.12 ± 0.66 kg/m^2^, respectively. All pens contained pine shavings as bedding (at approximately 6 cm depth), two galvanized tube feeders, and two water lines with nipple drinkers. Both feed and water were provided ad libitum. The corn-soy diets were prepared according to the nutritional specifications for conventional broiler chickens^35^ and were separated into three rearing phases: starter (day 1–22; 3000 kcal ME and 23% CP), grower (day 22–44; 3100 kcal ME and 21.5% CP), and finisher (day 44–53; 3150 kcal ME and 20% CP). House temperature started at 35 °C on day 1, and was gradually reduced to 21 °C on day 29, and remained at 21 °C until the end of the trial. The chickens were maintained on an artificial lighting program of 24L:0D in the first 4 days due to heat lamps, 20L:4D from day 5 to 7 of age, and 18L:6D until the end of the trial, with a light intensity of approximately 15 lx during light hours.

### Experimental design

**Figure 1:**
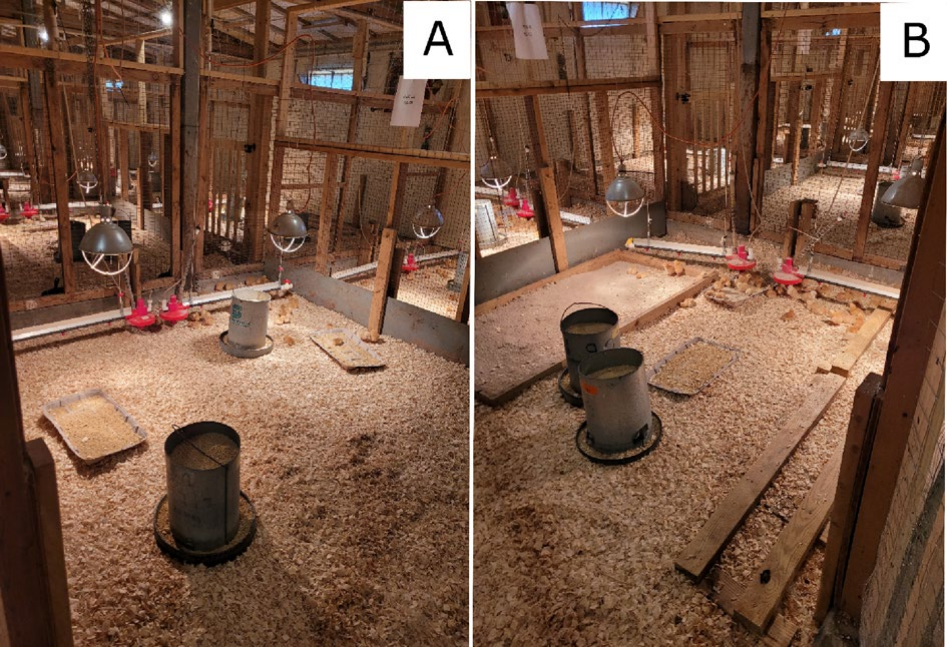
Chickens housed in two complexity environments. (**A**) Low-complexity, similar to commercial standards with feed, water and shaving; and (**B**) High-complexity, with permanent and temporary enrichments.

The trial consisted of a randomized block design of two environmental complexity treatments with six replicates each. The low-complexity control environment provided chickens conditions similar to commercial standards. The high-complexity environment provided chickens permanent and temporary enrichments. Permanent enrichments included a dust bath constructed from lumber (108 cm L × 91 cm W × 10 cm H) filled with playground sand (Quikrete, GA, USA) and two wooden platforms (488 cm L × 45.5 cm W × 7.5 cm H) in each pen, providing 19.5 cm linear perch space per bird (Fig. 1). Six temporary enrichments were rotated every 3 days, with two enrichments in each pen at one time. These enrichments included two plastic treat balls (Ethical Products, Inc., NJ, USA) filled with oats placed onto the litter paired with two bundles of string hung from the pen barrier. Half a cabbage hung at chicken height paired with alfalfa hay provided in two metal cage balls (20.3 cm diameter; Darice, OH, USA) placed on the litter. Two rectangular hanging mirrors (19 × 28 cm) paired with a handful of chicken scratch thrown into the litter (corn, wheat, milo, barley, oat, sunflower seed, and mullet; Manna Pro Products, MO, USA).

### Judgment bias procedure

At 24 days of age, 24 wing-banded chickens (n = 2/pen) were arbitrarily selected and gently marked on wings and legs with a livestock marker (All-Weather Paintstik, LA-CO Industries, Inc., IL, USA). These markings were reapplied as necessary throughout the experiment. The judgment bias procedure followed a 4-step process, including habituation, two training phases, and testing (see supplementary material for detailed procedures). The judgment bias test consisted of a multimodal approach using location (spatial) and color (visual) as cues. Prior to any task, two chickens per pen were placed in a holding pen of 4.37 m^2^. This had two functions, (1) it allowed the researcher to prepare the test arena for the judgment bias tasks after chickens were collected from the home pen, and (2) it allowed chickens to remain calm or calm down in a familiar environment comparable to the home pen, with a familiar conspecific, and with access to shavings, one feeder, and one water line. Food and water were provided ad libitum. One researcher performed all procedures.

#### Test arena

All procedures were performed in an arena made from plywood (91 cm L × 61 cm W) with yellow rubber interlocking mats (QC-Eb18N, Jiasheng, China) as flooring, two LED light bars, and a start box (30 cm L × 61 cm W) with access to the test area through a guillotine door (Supplementary Fig. S1). A camera (Teledyne Flir LCC, OR, USA) was placed overhead to record all judgment bias procedures. All tasks were conducted between 8:30 and 13:00 h.

#### Habituation

Chickens were first habituated to the judgment bias arena from 24 to 27 days of age (Supplementary Fig. S2). Chickens were habituated in pairs four times in the arena containing three arbitrarily-placed cardboard feed flats (5 × 5 cm) filled with dried mealworms and three empty transparent plastic bowls (120 ml; Ziploc®, S.C. Johnson & Son, Inc.). In the first round, chickens were placed directly into the test area with the start box door closed for 3 min while the observer was out of the chickens’ line of sight. In the second round, chickens were placed into the start box with the guillotine door open for 5 min while the observer was out of the chickens' line of sight. In the third and fourth rounds, chickens were placed in the start box with the door closed. The door was then opened, and chickens could move freely for 5 min while the observer remained in the chickens’ line of sight. Chickens were considered habituated when they consumed a mealworm during any habituation round. All chickens proceeded to the training phase.

#### Training

The training was divided into phase 1 and 2 and was performed when chickens were between 30 and 51 days of age. Chickens were trained in pairs to associate a color cue of 100% black (n = 6) or white (n = 6) and location right (n = 6) or left (n = 6) with approximately 10 mealworms as a reward stimulus. Rewarded cues and locations were balanced across treatments. The color cue paper (25 cm L × 12 cm W) and a plastic bowl (120 ml; Ziploc®, S.C. Johnson & Son, Inc.) of the same color were placed on or by the far wall of the arena, either at the far left or right depending on the color of the cue, with black rewarded cues always placed on the right and white rewarded cues always placed on the left. The opposite color and location represented the neutral stimulus, which consisted of a similar paper and bowl combination without a mealworm reward.

Training phase 1 was divided into two steps, A and B, and was response contingent. Depending on how chickens performed in the first step, they were assigned to step B or training phase 2 (Supplementary Fig. S3). During training phase 1A, paired chickens were presented with the positive cue and were required to peck it. Each training round lasted 3 min per pair, with 6 training attempts of 30 s per round. The chickens were given 30 s to respond to each attempt's positive cue. After each 30 s attempt, the observer gently picked up the chicken and placed it back into the start box to set up the arena for the next attempt. Unsuccessful attempts were followed by the observer immediately shaking and tilting the container. These rounds were repeated until chickens were considered learned (peck reward cue 5 out of 6 training attempts within a single round). If one or both chickens did not succeed in pecking at least 2 out of 6 times within the first round, they went to step B.

Training phase 1B (shaping) was used to allow those chickens a greater chance to reach the training criterion (Supplementary Fig. S4). During 3 rounds, a shaping procedure (Supplementary Fig. S5) was used until the rounds’ learning criteria were met. After all rounds, the chickens that pecked cue 2 out of 6 training attempts within a single round were moved back to step A and remained until chickens were considered learned. The chickens that pecked cue 5 out of 6 within a single round were moved to training phase 2. One pen (2 chickens) from the low-complexity treatment was removed from the test because the animals did not meet with learning criterion.

Training phase 2 (discrimination) began with 11 pens (n = 22, 2 chickens/pen). Positive and neutral cues were presented individually according to a pseudorandomized order, with no more than two of either cue presented consecutively (Supplementary Fig. S6). Each training session lasted 180 s per group with six training attempts of 30 s. Phase 2 continued until at least one chicken met the training criterion (chickens must peck positive cue 100% of the time and not peck neutral cue 100% of the time they were presented into a single round). Pairs from 9 out of 11 pens (n = 18 chickens) met the training phase 2 learning criterion. In both pairs remaining, one chicken in each pair met the learning criterion. These two pairs moved on to the testing phase, but only the performance of chickens that met the learning criterion was recorded. All training took 13 days distributed across 3 weeks.

#### Testing

The testing phase occurred in week 8 (days 52 and 53). All 11 pens (pairs) that advanced to testing (n = 20 chickens) were tested four times over 2 days. During testing, the Positive (P), Neutral (N), and three new, ambiguous cues, Near Positive (NP), Middle (M), and Near Neutral (NN), were individually presented with intermediate colors and at intermediate locations, 75% black (near right), 50% black (middle), and 25% black (near left) using a pseudorandomized order (Supplementary Fig. S7). Each testing session lasted a maximum of 7 min, with 1-min attempts for the chickens to approach each presented cue (in total, 28 attempts/pair). The first and last attempts in a testing session were always rewarded for maintaining motivation throughout the test. The researcher live-recorded frequency and latency to approach and proportion of chickens pecking cues. When one or both chickens did not approach or peck the cue, a maximum latency of 60 s was recorded.

### Lameness (gait)

The gait of all tested chickens was assessed by a trained observer when chickens were 53 days of age. A six-point scale was applied to classify gait scores^36^: score 0 normal walking, score 1 the chicken moves fast, but a slight walking deficiency is observed, score 2 the chicken moves fast, but there is a significant walking deficiency, score 3 the chicken moves fast, but it presents an important walking deficiency, score 4 the chicken moves with a serious difficulty, and score 5 the chicken barely moves and often uses its wings during locomotion.

### Chronic stress

After the testing phase (day 53), three wing feathers were cut from all tested chickens to assess feather corticosterone concentrations as described in^37^. Feather weight was recorded. Feathers were minced into pieces (< 5 mm), then HPLC-grade methanol (10 mL) was added. The samples were placed in a sonicating water bath at room temperature for 30 min, incubated in a shaking water bath at 50 °C overnight, and methanol was separated from the feather material through a vacuum filter. After that, the original sample vial and filtration material were washed twice with 2.5 mL of methanol and added to the original methanol extract to avoid losing any corticosterone from the sample. The methanol extract was placed in under at the fume hood until the methanol had evaporated. The extracted residues were reconstituted in a small volume of the phosphate buffer system. A commercially available ELISA (Cayman Chemical Company, MI, USA) was performed according to the manufacturer protocol to quantify the concentration of feather corticosterone by mm and mg of feather material.

### Behavioral observations

A tonic immobility test and attention bias test were performed in order to categorize chickens as fearful or anxious. The tonic immobility test assessed fearfulness^38,39^ and the attention bias test assessed anxiety^39,40^ when birds were 63 and 64 days of age, 10–11 days after the JBT testing phase was completed. Both tests lasted 300 s, and birds were categorized by relative fearfulness and anxiety using the median latency to righten and latency to begin feeding, respectively. When latencies were higher than the median, chickens were categorized as fearful or anxious. When latencies were below the median, chickens were categorized as fearless or calm (Supplementary Table S1).

#### Tonic immobility test

Chickens were subjected to a tonic immobility test, following the methodology described by^39^. Chickens were individually placed on their backs in a V-shaped wooden cradle with their heads hanging over the edge. The observer induced tonic immobility by placing two fingers on the bird’s sternum and applying gentle pressure while covering the bird’s head with the other hand for 15 s. Thereafter, the observer removed both hands. If a chicken rightened within 10 s after releasing, tonic immobility was reattempted. If tonic immobility was not induced after three attempts, the chicken was returned to their pen with their latency to righten recorded as 0 s.

#### Attention bias test

The attention bias test (ABT) was performed, following the methodology described by^39^. A square arena (125 cm W × 125 cm L × 91.4 cm H) was used with pine shavings on the floor, and a feeder containing commercial feed and mealworms. After each pair of chickens was placed in the arena, together with a third arbitrarily selected bird from the same pen as described by^39^, an 8 s conspecific alarm call was played to elicit a vigilance response. Latency to begin feeding (s), latency to first vocalization (s), and occurrence (yes/no) of vigilance behaviors in the first 30 s following the first alarm call (visibly stretching neck, looking around, freezing, and erect posture)^5,40^ were recorded. The alarm call wasreplayed and latency to resume feeding was recorded depending on birds’ responses, as described by^39^.

### Statistical analysis

Data were analyzed in SAS Studio 3.8 (SAS Institute Inc., Cary, NC, USA). The variance homogeneity was assessed using Levene’s test and data residual normality was verified by the Shapiro–Wilk’s test. The distribution of data residuals and subsequent statistical approaches are shown in Table 1. Even though residuals were not normally distributed for latency to approach (Table 1), the use of mixed-effects models is appropriate as these are largely robust even to quite severe violations of model assumptions such as the residuals’ distribution^41^. Model assumptions for skewness (0.136) and equal group variances (Levene’s test P = 0.648) were met^42,43^. Generalized linear mixed models (GLIMMIX) were followed by F-tests or Tukey’s multiple comparison tests. Correlations between chronic stress and fearfulness, chronic stress and anxiety, and anxiety and fearfulness were assessed using Pearson’s correlation analysis with the CORR procedure. Associations were considered significant at P < 0.05 and a trend at P < 0.1.

**Table 1:** Statistical approaches per measurement, with response variable tested, distribution of data residuals, statistical test used, predictors that were assessed, and random variables that were included in the model.

Analysis	Measurement	Response variable (unit)	Distribution of data residuals	Test	Predictors tested in the model	Random variables included
Univariate	Judgment bias training	Training rounds (n)	Other	Wilcoxon rank-sum	Treatment	n/a^2^
					Fear	
					Anxiety	
					Chronic stress	
	Judgment bias test	Latency to approach (s)	Other	GLIMMIX^1^	Gait	Pen (bird ID), round, treatment, cue type, reward color/side
					Fear	
					Anxiety	
					Chronic stress	
					Reward color/location	Pen (bird ID), gait, round
		Chickens that pecked cues (%)	Binary		Gait	Pen (bird ID), round, treatment, cue type, reward color/side
					Fear	
					Anxiety	
					Chronic stress	
					Reward color/location	Pen (bird ID), gait, round
	Tonic immobility	Latency to righten (s)	Other	Wilcoxon rank-sum	Treatment	n/a
	Attention bias	Latency to begin feeding (s)				
		Latency to first vocalization (s)				
		Latency to resume feeding (s)				
		Vigilance behaviors (%)				
	Gait	Score (0–5)				
	Chronic stress	Feather corticosterone concentration (µg/mg)	Normal	GLIMMIX		Pen (bird ID)
Multivariate	Judgment bias test	Latency to approach (s)	Other	GLIMMIX	Treatment, cue type, and their interaction	Pen (bird ID), round, gait, cue color/side
		Chickens that pecked cues (%)	Binary			

^1^Generalized linear mixed models.

^2^Not applicable.

## Results

### Judgment bias test

#### Habituation

One pair of chickens from the low-complexity treatment did not eat mealworms throughout the 4 habituation rounds. Therefore, they were omitted from the experimental procedures and replaced with a new arbitrarily selected pair from the same pen. During habituation round 1, 25% of chickens consumed at least 1 mealworm (latency mean ± SD: 127 ± 17 s), 75% in round 2 (97 ± 24 s), 96% in round 3 (36 ± 11 s), and 100% in round 4 (18 ± 7 s).

#### Training

In training phase 1A (conditioning for reward cue), chickens from 6 (2 low-, 4 high-complexity pens) out of 12 pens met the learning criterion and proceeded to training phase 2 (Supplementary Table S2). The unsuccessful chickens from the other 6 pens (4 low-, 2 high-complexity pens) were moved to training phase 1B (shaping for reward cue).

Chickens from 5 pens (2 low-, 3 high-complexity pens) took between 2 and 7 rounds to meet the 1B learning criterion. Two chickens from a single low-complexity pen were omitted from the experimental procedures because they did not meet the phase 1B learning criterion after 7 rounds.

In training phase 2 (discrimination between reward and neutral cue), chickens from 9 out of 11 pens took between 1 and 7 rounds (median of 2 rounds/pen) to meet the learning criterion. One of two chickens from each remaining pen (1 low-, 1 high-complexity pen) met the criterion, while the other did not meet the learning criterion after 7 rounds. Complexity treatment, fearfulness, anxiety, and chronic stress did not impact learning success (number of rounds needed to meet training criteria) for any training phase or overall (Supplementary Table S2). By the end of training phase 2, 83% of chickens (20 out of 24) successfully met the learning criterion.

#### Testing

Fear, anxiety, chronic stress, and gait score did not impact JBT responses (latency to approach or proportion of chickens pecking cues; P ≥ 0.180). Test round did not impact the latency to approach (F3,287 = 0.54; P = 0.654) or proportion of chickens that pecked cues (F3,287 = 0.38; P = 0.766). Reward side (right and left) or color (black or white) did not impact the latency to approach (F1,188 = 1.08; P = 0.301) or proportion of chickens that pecked cues (F1,185 = 0.33; P = 0.564).

**Figure 2:**
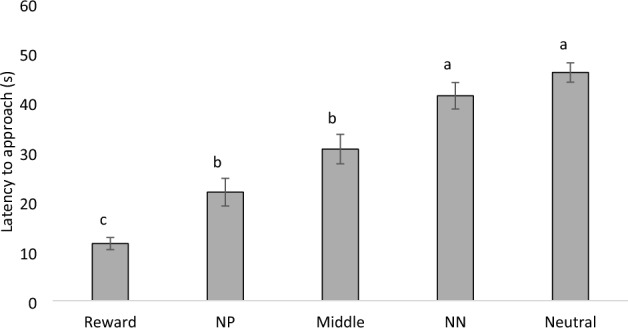
Mean (± SEM) latency to approach (s) all five cues (Reward; Near-positive [NP]; Middle; Near-neutral [NN]; and Neutral) in 4 rounds of the judgment bias test (n = 11 social pairs). Means with uncommon superscripts (^a–c^) differ at P < 0.001.

**Figure 3:**
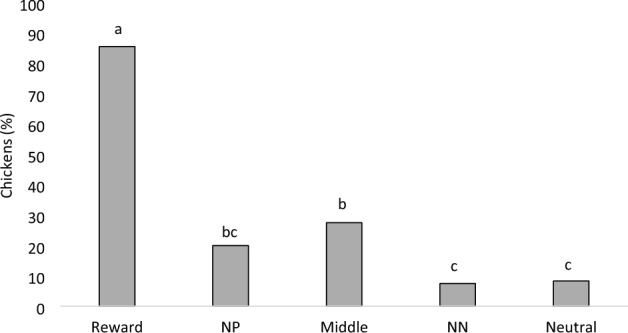
Proportion (%) of chickens that pecked all five cues (Reward; Near-positive [NP]; Middle; Near-neutral [NN]; and Neutral) in 4 rounds of the judgment bias test (n = 11 social pairs). Proportions with uncommon superscripts (^a–c^) differ at P < 0.001.

Regardless of treatments, chickens successfully discriminated between cues, as they approached the reward cue faster than near-positive (NP; P = 0.008), middle (P < 0.001), near-negative (NN; P < 0.001), and neutral (P < 0.0010 cues, they approached the NP cue faster than NN (P < 0.001) and neutral cues (P < 0.001), and approached the middle cue faster than the NN (P = 0.002) and neutral (P < 0.001) cues (F4,287 = 78.16; P < 0.001; Fig. 2). Similarly, more chickens pecked the reward cue compared to NP (P < 0.001), middle (P < 0.001), NN (P < 0.001), and neutral (P < 0.001) cues, and more chickens pecked the middle cue compared to NN (P = 0.016) and neutral (P = 0.007) cues (F_4,287_ = 46.33; P < 0.001; Fig. 3).

**Figure 4:**
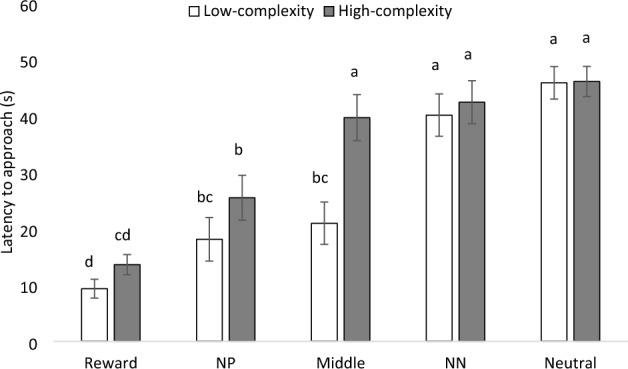
Mean (± SEM) of latency to approach (s) all five cues (Reward; Near-positive [NP]; Middle; Near-neutral [NN]; and Neutral) in 4 rounds of the judgment bias test by complexity treatment (n = 11 social pairs). Means with uncommon superscripts (^a–d^) differ at P < 0.05.

An interaction effect between treatment and cue type was found for latencies to approach cues (F_4,287_ = 3.56; P = 0.029, Fig. 4). Chickens from the low-complexity treatment approached the middle cue faster than chickens from the high-complexity treatment (P = 0.014), but no differences between complexity treatments were found for the reward (P = 0.992), NP (P = 0.951), NN (P = 0.660), and neutral (P = 0.926) cues. Chickens from the low-complexity treatment tended to approach cues faster than those from the high-complexity treatment (F_1,287_ = 3.13; P = 0.077). More chickens in the low-complexity treatment (45%) tended to peck cues than chickens in the high-complexity treatment (35%, F_4,287_ = 3.22; P < 0.073). No interaction between complexity treatment and cue type was found for the proportion of chickens pecking the cues (F_4,287_ = 2.15; P = 0.174).

### Lameness (gait)

The environmental complexity treatment did not affect gait score (χ^2^ = 0.20, P = 0.888), with 66.7% of chickens from the low-complexity treatment receiving a score 0 and 33.3% a score 1, and 63.6% of chickens from the high-complexity receiving a score 0 and 36.4% receiving a score 1.

### Behavioral observations

The environmental complexity treatment did not impact latency to righten in the tonic immobility test (Supplementary Table 3). In the attention bias test, more chickens in the low-complexity treatment performed vigilance behaviors such as looking around and freezing compared to chickens in the high-complexity treatment (Supplementary Table 3). Complexity treatment did not impact the proportion of chickens performing neck stretching, or latencies to first vocalization, to begin feeding, or to resume feeding (Supplementary Table 3). Erect postures were not observed during the attention bias test.

### Chronic stress

**Figure 5:**
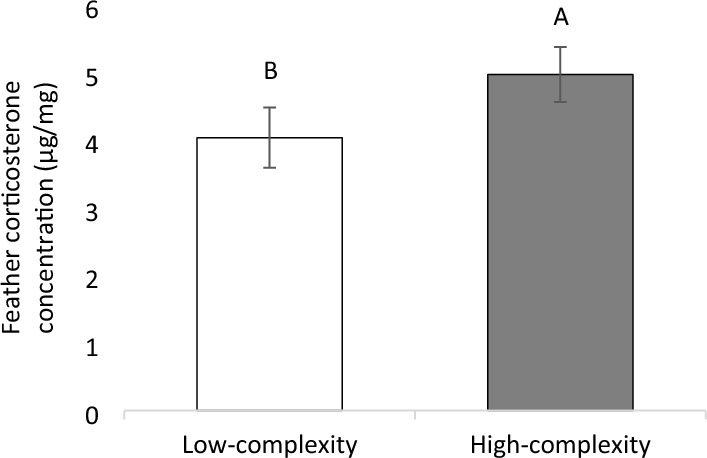
Least square mean estimates (± SEM) of feather corticosterone concentrations for chickens from low- or high-complexity treatments (n = 22 chickens). Means with uncommon superscripts (^A-B^) differ at P < 0.1.

Feather corticosterone concentrations tended to be higher in chickens from the high-complexity treatment than those from the low-complexity treatment (F_1,19_ = 3.63; P = 0.071, Fig. 5). No correlations were found between chronic stress and fearfulness (r = 0.310, P = 0.161), chronic stress and anxiety (r = 0.154, P = 0.494), or anxiety and fearfulness (r = 0.333, P = 0.123).

## Discussion

This is the first study to apply a social-pair training and testing approach in a judgment bias task. We inferred the affective state of slow-growing broiler chickens housed in low- or high-complexity environments from behavioral responses in the JBT. In addition, we evaluated the effects of fearfulness, anxiety, and chronic stress (feather corticosterone concentration) on JBT training and testing performance. During 13 days of training across 3 weeks, 83% of chickens were successfully trained to discriminate between multimodal reinforced cues. Chickens showed a generalization gradient response in the JBT, demonstrating that chickens successfully learned the discrimination task^3,5,12^. All chickens approached and pecked ambiguous cues close to the reward cue more quickly or more often than cues close to the neutral cue, without showing a color or side bias. This suggests a greater expectation of a reward for ambiguous cues near the reward cue. Based on these findings, a social-pair JBT approach can be used as a tool to infer affective states in slow-growing broiler chickens.

Complexity treatment, fearfulness, anxiety, and chronic stress did not impact the number of rounds chickens needed to meet the learning criterium for each training phase. Training chickens in pairs may have attenuated the effects of social isolation and novelty inherent to the JBT environment, especially for chickens with negatively valenced affective states. Studies using individual training approaches showed that stress and fearfulness negatively impacted laying hens’ and fast-growing broilers’ training performance in cognitive tests^19,26,44^. Broilers that were stressed by social isolation showed impaired spatial memory learning compared to a control group^19^. The authors argued that chronically stressed broilers were less capable of coping with negative conditions associated with the test (isolation and novelty)^45,46^, sensitizing them to respond to future stressful events (more testing) and provoking a shift in cognitive functions away from spatial learning and toward a stress response^19,47^. Similarly, rats and mice chronically stressed by social isolation showed impaired spatial learning in a water maze test compared to a control group^48,49^. Fearful rats made more side errors in Y-maze^50^ and water maze tasks^51^, while anxious rats showed poor learning performance in a water maze task^52–55^ compared to their control group. Furthermore, fearful, stress-sensitive laying hens developed side biases during JBT training^26,44^. The authors argued that fearful hens use a rigid response strategy during early learning phases by choosing a specific side repeatedly irrespective of success, indicating cognitive inflexibility^26,44^. In the current study, no such effects of fearfulness, anxiety, or chronic stress were observed on learning success or test responses.

The social-pair testing approach may have attenuated the negative effects of negatively valenced fearfulness, anxiety, and chronic stress. During testing, broilers experienced social support from a flock mate, which could increase their motivation to perform tasks. Broilers have a strong motivation for social reinstatement and chickens in natural settings live in relatively small, highly social groups^56–58^. In line with that, laying hens exhibited less fear-related behaviors when undergoing an open field test^59^ and fast-growing broilers performed better in an attention bias test when tested with two conspecifics compared to being tested alone^39^. These results suggest that chickens benefit from social support in testing environments that require learning or attention^15^. Our social-pair testing approach could have been especially beneficial for fearful, anxious, or chronically stressed animals, reflected in their similar learning performance compared to birds that were considered fearless, calm, or experiencing less chronic stress.

The learning success rate (20/24 chickens) in this study was greater than reported in earlier studies using an individual approach for fast-growing broilers and laying hens^5,7,9,12,13,60^, but lower than reported in^14^. Days needed to train birds were comparable to or faster than most other JBT studies. Training took between 10 and 30 days for fast-growing broilers, with low learning success rates (between 25 and 51%)^5,9^, and training took between 13 days and 8 weeks for laying hens, with better learning success rates (between 62 and 100%)^6,7,12,13^. However, all genetic strains differed from the strain used in the current study, which could influence the result.

We theorize that social facilitation improved chickens’ learning ability. Social learning helps chickens to decide what to eat and avoid^15^. Aversive behavior of one chicken towards a food item will result in consistent avoidance of that food item in an observing chicken^61^. As training phases 1A and 1B required birds to peck a reward cue with a food item, social learning (one bird observing another) could have contributed to chickens learning to peck the reward cue containing attractive food items. Furthermore, the paired approach could have facilitated spatial memory development and cue discrimination ability in training phase 2, as young chickens can locate hidden objects due to their developed spatial memory^62^, which allows them to learn from conspecifics through observation^63^.

The benefit of a social training approach could differ depending on genetic strain, yet in the current study only a slow-growing broiler strain was tested. Inherent stressors associated with JBT training and testing are the frequent handling by and close proximity with humans, plus repeated removal from home environments and flock mates, which could result in chronic stress^64^. These JBT procedures may be more distressing to slow-growing broilers than fast-growing broilers, as slow-growing broilers are more reactive to human interaction^65–69^. The improved training success compared to previous studies could suggest that the presence of a conspecific alleviated some of these negative experiences, thus a social approach may be especially beneficial for slow-growing broiler chickens. As chickens are a social species, this benefit is expected for other genetic strains too. Further research on social approaches in other genetic strains can confirm this.

Chickens from the low-complexity treatment were faster to approach all cues and the middle cue compared to chickens from the high-complexity treatment. Furthermore, more chickens from the low-complexity treatment tended to peck cues than those from the high-complexity treatment. These differences indicate that chickens from simple environments were more optimistic than chickens from enriched environments, in contrast with our hypothesis. One explanation could be related to the environmental enrichment used has been inappropriate for slow-growing broiler chickens and negatively impacted their affective state. Our enrichment strategy was to provide a complex and varied environment, maintaining environmental novelty and providing resources to fulfill highly-motivated behavioral needs. This highly complex environment effectively improved emotions and affective states in fast-growing broiler chickens^5,39^. However, slow-growing broiler chicken strains are more active and interact more with conspecifics and the environment than fast-growing strains^70,71^, which could have negated the potential benefit of the chosen enrichment items. If the enrichments were unsuited for slow-growing broilers, they might have elicited frustration or other negative emotions, resulting in negative affective states. In line, a highly-complex environment increased chronic stress parameters in mice and corvids compared to animals kept in barren environments^72–74^. Further supporting this theory and previous research findings, the chickens from the high-complexity treatment tended to show an increased chronic stress response compared to chickens in the low-complexity treatment. The increased chronic stress response in high-complexity chickens could in part be due to the increased human-animal interactions associated with providing temporary enrichments, since slow-growing broilers are more reactive to human interaction than fast-growing strains^65–69^. Alternatively, the novelty of these enrichments, assuming that novelty was maintained, might have increased birds’ arousal and thus increased corticosterone deposition in feathers. Increased arousal can increase circulating corticosterone concentrations, also when animals experience a positively valenced emotion such as pleasure, excitement, and winning^75–77^. As the majority of research on environmental enrichments for broiler chickens is focused on fast-growing strains^78^, we recommend further research assessing slow-growing broilers’ preferences for environmental enrichments.

In the current study, complexity treatments did not affect gait score, with gait being perfect or slightly deficient for all birds assessed. In line, slow-growing broilers generally have good walking ability^66,79^. Latency to approach cues and proportion of chickens pecking cues were not impacted by gait, in line with fast-growing broilers in a JBT^5^. These results suggest that observed differences in latencies and proportion of chickens approaching were reflecting a cognitive bias instead of physical limitations to approach the cues.

In order to avoid the effects of fear and anxiety tests on JBT responses, we performed these tests 10 days after the JBT testing phase. As life experiences shape behavioral responses and individual preferences over time^27^, the JBT procedure could have impacted the chickens’ behavioral responses on fear and anxiety tests. Repeated training and testing could have reduced fear towards humans as chickens habituated to repeated handling^80^. This study design did not allow us to assess a balanced sample of fearful, fearless, anxious, and calm chickens in both complexity treatments. In addition, our limited sample size may have reduced the statistical power to assess the effects of fearfulness, anxiety, and chronic stress on training performance. We recommend further research assessing fear, anxiety, and chronic stress with a larger sample size to confirm the lack of impact found in the current study. Furthermore, this study did not incorporate a control group to directly compare a social-pair JBT approach with an individual JBT approach due to time constraints. Yet, even without a direct comparison, the social approach seems to result in improved learning ability compared to training success when birds are tested individually^5,7,9,12,13,60^.

To conclude, this study is the first to show that a social-pair judgment bias training and testing approach can be used to successfully assess affective states in slow-growing broiler chickens, with no effects of fearfulness, anxiety, or relative chronic stress (based on feather corticosterone concentrations) on the chickens’ learning ability during training or testing. The judgment bias test in this study showed that slow-growing broilers housed in unenriched, low-complexity environments were more optimistic to receive a reward in an ambiguous situation than broilers from an enriched, high-complexity environment. We recommend further studies directly comparing individual and social-pair JBT approaches. Chickens from the high-complexity treatment tended to show an increased chronic stress response and a more negative affective state compared to the chickens in the low-complexity treatment, possibly related to the suitability of provided environmental enrichments.

## Supplementary Information

Supplementary Information.

## Data Availability

Data underlying this manuscript are made accessible through the Virginia Tech Data Repository at 10.7294/22155401.
