# Supplementary material for: Social-pair judgment bias testing in slow-growing broiler chickens raised in low- or high-complexity environments
Source: Sci Rep. 2023 Jun 9;13:9393. doi: 10.1038/s41598-023-36275-1 (PMC10256692; doi:10.1038/s41598-023-36275-1)
Supplement: Supplementary file 1 — Supplementary Information. [file 41598_2023_36275_MOESM1_ESM.docx]

**Supplementary information for:**

**Social-pair judgment bias testing in slow-growing broiler chickens raised in low- or high-complexity environments**

M. I. Lourenço-Silva¹ ², A. Ulans², A.M. Campbell^2^ , I.C.L. Almeida Paz^1^ , L. Jacobs²*

^1^ Department of Animal Production and Preventive Veterinary Medicine, School of

Veterinary Medicine and Animal Sciences (FMVZ), São Paulo State University “Júlio de

Mesquita Filho” (UNESP), Botucatu, São Paulo, Brazil

^2^ School of Animal Sciences, Virginia Tech, Blacksburg, VA, 24061 USA.

*Corresponding author: jacobsl@vt.edu

**Table S1.** Mean estimate responses and proportions (%) of broiler chickens characterized as fearful or fearless in the tonic immobility test (s ± SEM; 0-300 s) and broiler chickens characterized as anxious or calm in the attention bias test (s ± SEM; 0-300 s)

| Tonic immobility test | | Characterized relative personality¹ | | |
| --- | --- | --- | --- | --- |
|  |  | Fearful | Fearless | n |
| Latency to righten (s) | | 165.4 ± 26.7 | 39.4 ± 5.9 | 22 |
|  |  |  |  |  |
| Attention bias test | | Anxious | Calm | n |
| Begin feeding (s) | | 174.4 ± 27.9 | 19.6 ± 5.9 | 22 |
| First vocalization (s) | | 8.8 ± 2.8 | 18.9 ± 10.5 | 19 |
| Resume feeding (s) | | 26.4 ± 8.9 | 25.4 ± 7.6 | 16 |
| *Vigilance behaviors* |  |  |  |  |
| Erect posture (% of chickens) | | 0.00 | 0.00 | 22 |
| Neck stretching (% of chickens) | | 12.50 | 0.00 | 22 |
| Looking around (% of chickens) | | 75.00 | 27.27 | 22 |
| Freezing (% of chickens) | | 50.00 | 0.00 | 22 |

¹Behavioral characterizations were done based on median scores of latency to righten in the tonic immobility test (fearfulness) and latency to begin feeding in the attention bias test (anxiety).

**Table S2**. Number of successful chickens (n) and JBT training rounds (mean ± SD) needed to meet the learning criteria by complexity treatment, personality trait, and chronic stress categorization.

| Training phase | 1A^1^ | | 1B^2^ | | 2^3^ | | Total | | Statistical test and *P*-value^4^ |
| --- | --- | --- | --- | --- | --- | --- | --- | --- | --- |
|  | Round n | Bird n | Round n | Bird n | Round n | Bird n | Round n | Bird n |  |
| *Complexity treatment* | | | | | | | | | |
| Low-complexity | 2 ± 1 | 4 | 2 ± 1 | 8 | 2 ± 1 | 9 | 5 ± 2 | 9 | χ² = 1.00, *P* = 0.317 |
| High-complexity | 2 ± 1 | 8 | 3 ± 1 | 4 | 2 ± 2 | 11 | 4 ± 2 | 11 |  |
| *Personality trait* | | | | | | | | |  |
| Fearful | 2 ± 1 | 7 | 3 ± 1 | 5 | 1 ± 1 | 10 | 4 ± 2 | 10 | χ² = 1.69, *P* = 0.193 |
| Fearless | 1 ± 1 | 5 | 2 ± 1 | 7 | 2 ± 2 | 10 | 5 ± 3 | 10 |  |
| Anxious | 2 ± 1 | 7 | 3 ± 1 | 5 | 1 ± 1 | 10 | 4 ± 3 | 10 | χ² = 0.53, *P* = 0.467 |
| Calm | 1 ± 1 | 5 | 2 ± 1 | 7 | 2 ± 2 | 10 | 5 ± 2 | 10 |  |
| *Chronic stress* | | | | | | | | | |
| Low-stressed | 1 ± 1 | 7 | 3 ± 0 | 2 | 2 ± 2 | 9 | 4 ± 3 | 9 | χ² = 0.72, *P* = 0.397 |
| High-stressed | 2 ± 1 | 5 | 2 ± 1 | 6 | 2 ± 1 | 11 | 5 ± 2 | 11 |  |

^1^Conditioning for reward cue

^2^Shaping for reward cue

^3^Discrimination between reward and neutral cue

^4^Univariate analysis for predictors (treatments, personality traits, chronic stress categorization) on total learning success (n rounds needed to reach learning criteria)

**Table S3.** Least squares mean estimates (± SEM) and proportions (%) of broiler chickens from low- or high-complexity treatments in the tonic immobility test (0-300 s) and the attention bias test (0-300 s)

| Personality trait test responses | Complexity treatments | | | Test statistic and P-value for complexity effect |
| --- | --- | --- | --- | --- |
|  | Low-complexity | High-complexity | Bird n |  |
| Tonic immobility test | | | | |
| Latency to righten (s) | 113 ± 33 | 84 ± 19 | 22 | χ² = 0.31, P = 0.575 |
| Attention bias test | | | | |
| First vocalization (s) | 11 ± 3 | 15.2 ± 9 | 19 | χ² = 0.51, P = 0.476 |
| Begin feeding (s) | 128 ± 35 | 70 ± 26 | 22 | χ² = 2.31, P = 0.128 |
| Resume feeding (s) | 25 ± 9 | 26 ± 7 | 16 | χ² = 0.01, P = 0.957 |
| Erect posture (% of chickens) | 0 | 0 | 22 | n/a^1^ |
| Neck stretching (% of chickens) | 11 | 0 | 22 | χ² = 1.11, P = 0.292 |
| Looking around (% of chickens) | 77 | 20 | 22 | χ² = 6.01, P = 0.014 |
| Freezing (% of chickens) | 44 | 0 | 22 | χ² = 5.33, P = 0.021 |

^1^No statistical analysis was performed because behavior was not observed

**Figure S1.** Judgment bias test arena. Chickens were placed in the start box prior to all training or testing sessions. Colored cues and associated containers (black or white) were placed at either reinforced location (left or right). During testing, three additional ambiguous-colored cues were placed at intermediate locations (near positive, middle, near neutral). During all training and testing phases, cues were individually presented.

**
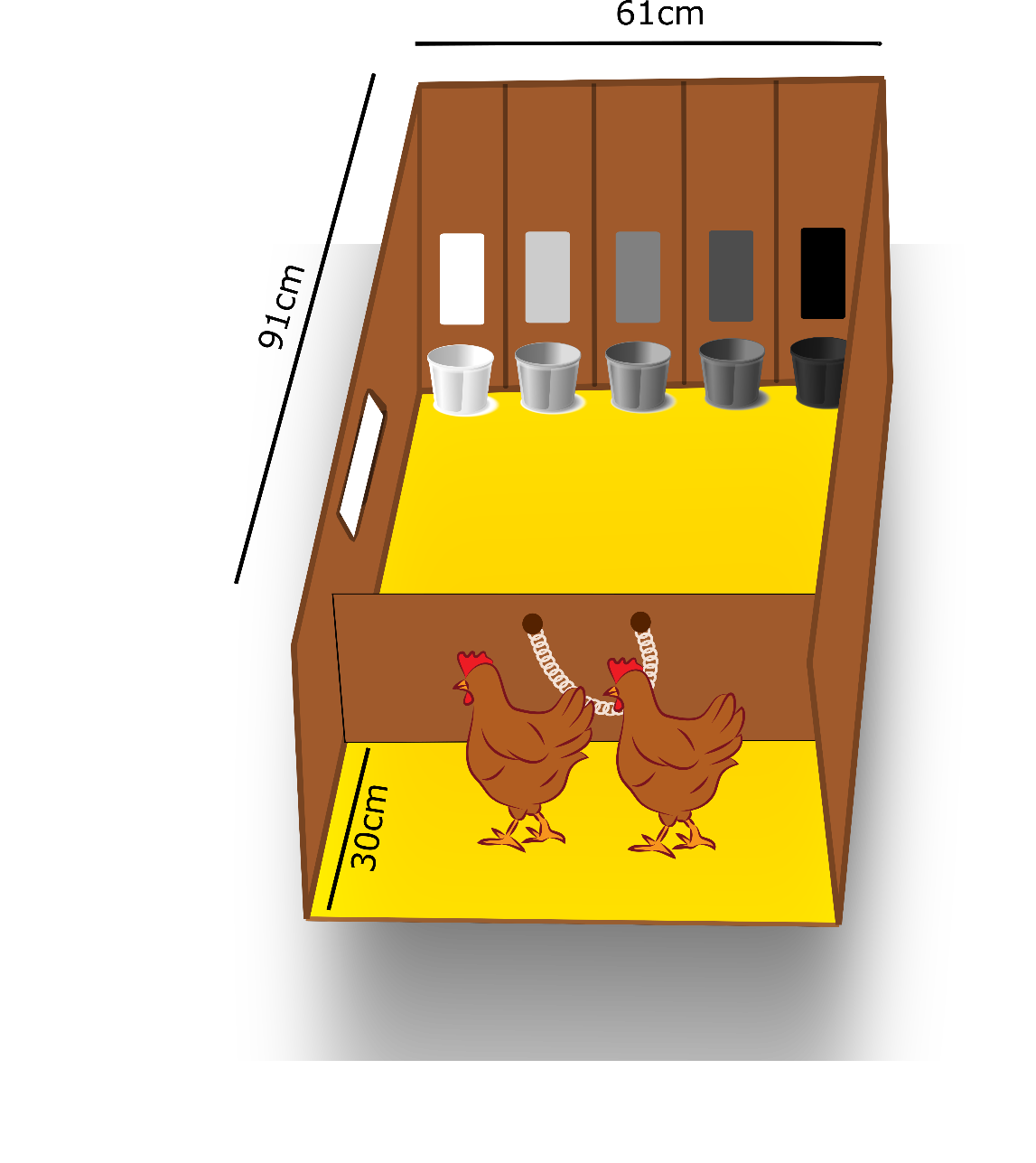
**

**Figure S2.** Diagram of the judgment bias habituation phase protocol (n = 24 chickens). All chicks undergo 4 habituation rounds. Blue boxes represent habituation phase methods, white boxes represent bird responses. Adapted from ^59^.

**Figure S3.** Diagram of the judgment bias training phase 1A protocol for round 1 (n = 24 chickens). Blue boxes represent training phase 1A methods, white boxes represent bird responses. Adapted from ^59^.

**Figure S4.** Diagram of the judgment bias training phase 1B protocol (shaping) (n = 24 chickens). Blue boxes represent training phase 1B methods, white boxes represent bird responses. Adapted from ^59^.

**Figure S5.** Diagram of the shaping procedure that was applied in each round of training phase 1B (n = 24 chickens). Learning criteria were orient (round 1), approach (round 2, or peck cue (round 3). Chicks will remain in this phase until successful, or the birds will be excluded from the trial after 7 rounds. Blue boxes represent shaping procedure methods, white boxes represent bird responses.

**Figure S6.** Diagram of the judgment bias training phase 2 protocol (n = 22 chickens). Blue boxes represent training phase 2 methods, white boxes represent bird responses. (A) Chicks are presented with a reward cue. (B) Chicks are presented with a neutral cue. Adapted from ^59^.

**Figure S7.** Diagram of the judgment bias testing phase protocol (n = 20 chickens). Blue boxes represent testing phase methods, white boxes represent bird responses. Each round has seven 1-minute attempts with reference cues (reward and neutral) and ambiguous cues (Near-positive [NP]; Middle; Near-neutral [NN]) being presented individually.

NO

YES

Allow to consume mealworms/investigate bowl for 5-10 sec, then remove chicks from the arena. Chicks are placed in start box or home pen

**Testing procedure**

Chick pairs are presented with reference (reward and neutral) and ambiguous (NP, middle, and NN) cues in a pseudorandomized order

ATTEMPT 1, 7 - REFERENCE CUE

Chicks are presented with reward cue

ATTEMPT 3, 5 - REFERENCE CUE

Chicks are presented with reward/neutral cue

ATTEMPT 2, 4, 6 - AMBIGUOUS CUE

Chicks are presented with NP, Middle, or NN cue

Place chicks back in start box. Next cue presented according to pseudorandomized order

Chicks approach and peck cue within 1 minute
